# Navigating the storm: Ethical consideration to climate justice and sustainable health equity in Africa

**DOI:** 10.1016/j.joclim.2025.100465

**Published:** 2025-08-05

**Authors:** Adetayo Emmanuel Obasa

**Affiliations:** Registrar Research Support Office, Research and Internationalisation, Development and Support Division, Faculty of Medicine and Health Sciences, Stellenbosch University, Cape Town, South Africa

**Keywords:** Climate Change, Climate Justice, Ubuntu, Health Disparities, and Vulnerable Population

## Abstract

Africa bears a disproportionate burden of climate change, despite contributing minimally to global greenhouse gas emissions. This article explores the ethical imperatives of addressing climate-induced health disparities in Africa, particularly through the lens of climate justice and the African philosophy of ubuntu*.* In Sub-Saharan Africa, approximately 700 million people live on less than $2.15 per day, falling below the extreme poverty line. Low-income communities disproportionately suffer from the negative impacts of climate change. These populations face a multifaceted crisis that requires clear, ethical, and specific solutions tailored to their unique needs. Drawing on theories of distributive, corrective, and participatory justice—as well as Rawlsian and utilitarian ethics—the paper critiques current global mitigation and adaptation strategies for their failure to adequately support African priorities. It further advances the case for integrating grassroots participation, community-based solutions, and inclusive governance. By prioritizing health equity, environmental justice, and ethical policymaking, the article proposes practical recommendations that align short-term needs with long-term sustainability. This article charts a pathway toward just and inclusive climate policies by arguing for a transformative response rooted in justice, solidarity, and African agency—prioritizing the needs of the most affected while fostering resilience and sustainability for future generations.

## 1 Introduction

The World Health Organisation (WHO) has identified climate change as the greatest threat to humanity [1]. Africa’s unique position in this narrative has been characterized by its low contribution to greenhouse gas emissions (under 4 %) juxtaposed with its high vulnerability to climate impacts [2]. From the prolonged droughts in the Horn of Africa to rising sea levels affecting coastal regions like Mozambique, the continent endures some of the most severe climate-related consequences [3]. This stark disparity between contribution and vulnerability stresses the urgent need for climate justice [4].

Efforts to address these vulnerabilities have seen mixed progress. The Glasgow Climate Pact, established during the 26th Conference of the Parties (COP26), aimed to double adaptation finance by 2025 [5,6]. Similarly, COP27 in Egypt marked a significant milestone with the establishment of a loss and damage fund [7]. There are still concerns about the availability, accessibility, and timeliness of these funds [8]. These issues raise questions about whether such financial mechanisms will effectively address critical challenges like displacement crises, agricultural losses, and health infrastructure damage. At COP28, despite a strong focus on boosting climate finance for adaptation, progress remained inadequate [9]. Sub-Saharan Africa urgently requires adaptation finance to strengthen its healthcare systems, improve resilience, and enhance water management strategies [9].

The COP 29 proceedings underline Africa's acute vulnerabilities to climate change through targeted initiatives addressing energy efficiency, agricultural resilience, and health system integration. Prominent programs like the African Energy Futures Initiative and the Baku Harmoniya Climate Initiative emphasized region-specific challenges. These efforts align with the urgent call for adaptation finance in Sub-Saharan Africa to bolster healthcare systems, enhance resilience, and improve water management strategies, as recognized by global frameworks and United Nations directives [10].

Africa is already experiencing devastating climate events that directly impact public health. Countries such as Kenya endure prolonged droughts [11], while South Africa faces destructive “rain bomb” events [12]. Severe flooding in Nigeria [13] and recurring cyclones in Malawi, Mozambique, Madagascar, and Zimbabwe [14] further exacerbate these challenges. These events result in displacements, death, injuries, malnutrition, and increased incidence of infectious and chronic diseases, placing unsustainable burdens on fragile health systems and threatening healthcare infrastructure.

The cascading impacts extend beyond health. From 2014 to 2019, climate change drove a 0.88 percentage-point rise in severe food insecurity and a 2.24 percentage-point increase in moderate to severe food insecurity in Africa, already the most food-insecure region globally [[15], [16], [17]]. These findings confirm that the effects of climate change undermine livelihoods, social stability, and public health outcomes [18].

Africa’s disproportionate burden raises pressing ethical considerations, such as historical responsibility, intergenerational justice, and the duty of care owed by global actors [19]. Although the Global North bears a moral obligation to address Africa’s climate challenges due to its higher emissions contribution, this must not translate into a paternalistic approach that excludes meaningful African participation [20,21]. Instead, a just response requires African nations to be equal partners in decision-making processes. This includes moving from debt-inducing aid to unconditional grants and reparations, thereby advancing solutions rooted in inclusivity and genuine collaboration. Prioritizing health equity in climate action metrics ensures that health outcomes are explicitly tied to climate financing and mitigation efforts.

This paper adopts the climate justice framework to critically examine the ethical dilemmas surrounding Africa’s vulnerability to climate change. At the heart of climate justice is the principle of distributive justice, which advocates for the equitable distribution of resources and targeted support for Africa's most vulnerable populations. Equally significant is the concept of corrective justice, which addresses historical and persistent inequities through mechanisms such as debt relief, recognizing the structural imbalances that shape the continent's climate challenges. Ubuntu emphasizes interconnectedness, communal solidarity, and collective welfare, aligning closely with distributive and corrective justice principles. By situating the analysis within this framework, the paper highlights the imperative of fair, contextually appropriate solutions to the climate crisis in Africa, while advancing a collaborative and inclusive global response.

## 2 Impact of climate change events on health in Africa

Africa spans over 30 million square kilometres, making it the second largest continent, second only to Asia, which covers approximately 44.58 million square kilometres [22]. The increasing unpredictability of weather and climate conditions across Africa has caused significant disruptions to economic systems, human settlements, and infrastructure. Between 2000–2019, six African countries—Somalia, Zimbabwe, Lesotho, Eswatini, Niger, and Mauritania—were among the top ten countries that experienced extreme weather events [23]. Since the beginning of 2022, extreme weather events in Africa have impacted at least 19 million individuals and caused over 4000 fatalities [24].

Africa experienced 134 droughts, with 70 occurring in East Africa alone [23]. Millions in Somalia, Ethiopia, and Kenya are struggling to survive amidst severe water shortages, widespread hunger, insecurity, and conflict [3]. At the end of 2022, the horn of Africa experienced the longest and most severe drought in history [3]. This resulted in the deadliest drought in Somalia, with 43,000 deaths, while Uganda has experienced several droughts, recording 2465 fatalities [25]. Although it has been argued that the reported death toll may not fully represent the true impact of droughts due to underreporting, it is essential to consider these arguments within the broader context of the complexities associated with data collection during crises [3]. Accessibility to certain regions is limited and security challenges impede accurate data collection, and potentially lead to underreporting [3].

The most devastating floods in ten years have impacted several countries including Nigeria, Niger, Benin, Chad, Mali, and Cameroon resulting in >876 mortalities. In April 2022, landslides, and floods in the South African provinces of KwaZulu–Natal and Eastern Cape, resulted in the deaths of 459 people and the displacement of an additional 40,000 [24]. In 2023, the Democratic Republic of Congo and Rwanda experienced flooding that led to 574 fatalities. Flooding in urban areas in Africa is often attributed to poor drainage systems [26], while in rural areas, it is due to deforestation [27]

In 2023, Cyclone Freddy caused significant disruptions in Malawi, Mozambique, and Madagascar, with the death toll reaching 1434. Cyclone Freddy's most intense rainfall began impacting Malawi on March 11. Following these rains, Mozambique experienced a tenfold increase in cholera cases, with 123 cholera-related deaths reported in the country since late 2022. Although the extreme weather conditions contributed to a cholera outbreak and related deaths, it is essential to acknowledge that pre-existing challenges and vulnerabilities, such as inadequate water and sanitation facilities, have a significant role in the transmission of water-borne diseases [28]

Recently the World Health Organisation (WHO) confirmed that climate change is playing a pivotal role in the global upsurge in cholera due to increased droughts, floods, and cyclones [29]. Studies have shown that climate change is expected to decrease cyclone frequency albeit increase their intensity–without anthropogenic warning, droughts, floods, and cyclone would still occur [30,31]. Intense cyclones could have more damaging effects than a higher frequency of cyclones. Somalia has faced increasingly severe drought since 2014, with regularly below-average rainfall and consecutive rainfall failures for crop production in late 2016 and 2017. These unpredictable weather patterns have resulted in large-scale displacement and outbreaks of epidemics [32].

The disproportionate burden borne by Africa raises crucial ethical questions about justice, equity, and historical accountability. As the continent grapples with the consequences of extreme weather events, the intersection of health inequities and climate change highlights the urgency for ethical climate action. This action must prioritize both mitigation and adaptation strategies, with a focus on addressing the structural factors contributing to these inequities. These ethical considerations demand a deeper reflection on the global obligations of wealthier nations, particularly in rectifying the tragedy of the commons and its disproportionate impact on vulnerable populations.

## 3 Ethics in the eye of the climate change storm and events in Africa

Climate change has been referred to as a “perfect moral storm” because it simultaneously brings significant challenges to ethical action [33]. In the African context, the intersection of climate change and health presents three challenges for ethical considerations. The first challenge is that health inequities exacerbated by climate change represent a critical ethical issue. The ethical concern here lies in the responsibility to mitigate these inequities, which are caused by historical contributions to climate change, while acknowledging that health inequities themselves are unjust conditions resulting from these imbalanced responsibilities. Climate change amplifies these pre-existing inequities [34]. Countries like Angola, Burundi, Ethiopia, Kenya, Madagascar, Malawi, Tanzania, Uganda, and Zambia have experienced severe hunger due to weather extremes [35]. These present public health challenges and raises critical ethical questions about the obligations of international bodies—whether through funding, policy reforms, or reparative action—to address these injustices.

International commitments such as the United Nations Framework Convention on Climate Change (UNFCCC), the Intergovernmental Panel on Climate Change (IPCC) [36], and the WHO’s climate initiatives aim to establish frameworks for combating climate change. The persistent health inequities suggest that these approaches are either insufficient or inequitably applied. There is a pressing need for targeted, context-specific solutions, particularly to address localized impacts within the sub-Saharan African region. Global frameworks, such as the Paris Agreement, often emphasize broad, long-term goals. While these goals are essential, the importance of context should not be overlooked [37,38]. A context-specific health adaptation program would need to prioritize strengthening health infrastructure, improving access to clean water, and strengthening agricultural resilience in regions most affected by drought [39]. The current commitments frequently fail to effectively allocate resources to address these specific challenges. Hence, it is imperative that these nations be included in meaningful decision-making processes that enable them not only to contribute but also to drive agendas that reflect their unique priorities and challenges.

Existing international agreements often impose a global perspective that may not always align with local needs [40]. There is a significant focus on mitigation and adaptation finance in international agreements, but their implementation is frequently uneven and inconsistent [41]. Africa, as one of the lowest contributors to global emissions, requires that these agreements include corrective justice mechanisms that prioritize debt relief rather than loans or external aid perpetuating financial dependency. The failure of these mechanisms is exemplified by the slow pace of the Green Climate Fund, which has been unable to fulfil its mandate. While these international commitments have provided a sturdy foundation for advancing climate science and policy, they require recalibration to address persistent inequities in health outcomes [42,43]. There is a pressing need to move beyond traditional top-down strategies towards an inclusive, accountable, and context-specific bottom-up approach [44]. Such an approach, driven by the principle of climate justice, would emphasize greater engagement with local communities, scientists, and policymakers in Africa to design interventions that are locally appropriate and more likely to succeed [44].

Considering health inequities and climate justice through the prism of the ‘tragedy of the commons’ describes how individuals acting in their self-interest tend to overuse and deplete shared resources, harming the collective [45]. Applied to climate change, the "commons" in question is the global atmosphere—a shared resource that everyone relies on but is being overexploited by more industrialized nations, primarily in the Global North. Wealthier nations, responsible for most historical emissions, have disproportionately exploited the global commons (i.e., the atmosphere) for their economic benefit. They have consumed more than their fair share of the Earth's carbon budget, leading to a climate crisis that disproportionately impacts the Global South, particularly Africa [43]. The tragedy also lies in the failure to safeguard the commons by ensuring equitable climate mitigation and adaptation strategies. Despite the role of wealthier nations in driving the crisis, they are often reluctant to provide the necessary financial or technical support to the countries bearing the brunt of its impacts. As a result, global climate efforts represent a tragedy of insufficient collective action, where powerful nations are reluctant to contribute their fair share to the solution.

Climate justice reframes the tragedy of the commons by emphasizing ethical and equitable resource management. It demands that powerful stakeholders contribute more significantly to global climate solutions and that the voices of marginalized groups—those historically excluded from resource governance—be actively included in decision-making processes [23,46]. This approach aims to prevent further exploitation of global resources and foster a more equitable world, where collective action is guided by principles of fairness, shared responsibility, and inclusivity [4].

The second challenge lies in balancing immediate short-term needs with sustainable long-term outcomes [41]. In Africa, this issue is particularly pressing in the context of addressing climate change [47]. Many African countries rely on carbon-intensive energy sources to support local livelihoods, especially in rural areas [47]. These energy sources are often indispensable for daily survival, whether for cooking, heating, or powering small businesses [48]. Overdependence on fossil fuels undermines both global health and public health. The combustion of fossil fuels leads to immediate and medium-term impacts on air quality and contributes to long-term consequences associated with climate change [44]. This reliance on fossil fuels and other unsustainable practices can significantly hinder efforts to achieve long-term climate resilience and improved health outcomes [49], [50], [51]. The ethical dilemma arises in navigating the tension between the short-term necessity of fuel access and the long-term imperative for environmental sustainability, particularly as it intersects with the economic development and well-being of vulnerable populations.

According to the World bank, approximately 648 million people live on less than $2.15 a day [52]. Their lived reality includes increased adverse weather conditions that exacerbate food and water security for these households [[11], [12], [13], [14],^47^]. The ethical dilemmas surrounding climate change must recognize that, while these communities have minimal emissions footprints, they face significant health, and livelihood risks due to the climate crisis. Discussions on sustainability cannot be separated from the economic realities of these populations, who rely on affordable, often unsustainable energy sources to meet their basic needs [53]. This highlights the moral imperative to prioritize the needs of these vulnerable groups when crafting climate and sustainability policies.

The tension between long-term sustainability goals and short-term economic survival is fraught with tension in Africa. Many African nations are reliant on fossil fuels for energy, hindering efforts to improve public health and environmental sustainability [49,54]. Algeria, Nigeria, Morocco, South Africa, and Egypt—often referred to as Africa's "Big Five"—are the continent's largest consumers of fossil fuels for energy generation [49]. Access to affordable fuel is essential for low-income households, as it directly affects their ability to cook meals, access clean or boiled water, and operate small businesses [50]. The continued reliance on fossil fuels not only contributes to future emissions but also poses significant health risks, particularly through indoor air pollution from biomass and kerosene usage, which has been linked to respiratory diseases and premature deaths [55,56]. The ethical challenge lies in ensuring energy access for the poor without further entrenching them in cycles of environmental and health risks. Addressing these competing needs requires innovative solutions that reconcile short-term relief with sustainable, long-term outcomes.

One way to navigate this balance is to focus on human-centred solutions that prioritize both the health and economic well-being of vulnerable populations [57,58]. Renewable energy initiatives in countries such as Kenya, Ethiopia and Mozambique have provided off-grid solar energy solutions to rural communities [59]. These projects not only reduce emissions but also offer a clean and reliable energy source that enhances health outcomes by decreasing reliance on harmful biomass fuels [51], [60], [61]. These initiatives contribute to economic empowerment by enabling small-scale businesses to operate sustainably. When solutions are context-specific and carefully designed, these examples illustrate that sustainability and economic development can align, even in low-income settings, to meet the needs of the communities they serve.

In the African context, discussions on sustainability must centre on values deeply embedded in the lived experiences of those most affected by climate change. For many communities in low-income settings, sustainability is not an abstract concept but a matter of survival—manifesting directly through improved access to clean water, food security, and healthcare. For instance, rural communities in Cameroon often depend heavily on natural water sources, whose reliability is intricately tied to local temperature and rainfall patterns [55]. From an ethical perspective informed subtly by the philosophy of Ubuntu—which emphasizes interconnectedness, communal solidarity, and collective responsibility [56], climate justice should ensure sustainability goals directly correlate with immediate improvements in quality of life [62]. This Ubuntu-informed approach reframes sustainability discourse, shifting it from discussions of trade-offs towards opportunities for collective betterment. Addressing climate change thus becomes not only an environmental imperative but also an ethical opportunity to enhance health, economic resilience, and communal well-being among vulnerable populations. Sustainability initiatives in Africa, guided by values, must consequently extend beyond mere emissions reduction to actively improving the communal life quality and fostering resilience among those disproportionately affected by the climate crisis.

A third challenge is attaining environmental justice and health equity, particularly within the Global South context. Addressing environmental justice is essential for achieving health equity, as it involves tackling the fundamental determinants of health disparities by ensuring universal access to uncontaminated air, water, and soil, along with safe and healthy living environments for all communities [[59], [63], [64]]. In Nigeria, millions of citizens in rural areas lack access to electricity, with the burden of energy poverty falling disproportionately on these populations. The effects of environmental injustice are further exacerbated by socio-economic and political disparities [62]. The oil and gas industry, a key driver of Nigeria's economy, has contributed significantly to environmental pollution, land degradation, and social conflicts in the Niger Delta region [65]. Communities in oil-producing areas have experienced environmental injustices, including oil spills, gas flaring, and the loss of livelihoods [65].

The ethical complexity lies in balancing economic development—which is vital for national growth—with the social and environmental responsibility needed to protect vulnerable populations. Although the Nigerian economy benefits from the exploitation of oil and gas resources, local communities pay the price through environmental degradation and health hazards. The exclusion of these communities from decision-making processes around resource extraction presents a procedural injustice, as they are deprived of a voice in matters directly affecting their environment and well-being. The ethical question then becomes: how can sustainable development be pursued in a way that does not perpetuate health inequities and environmental harm?

Addressing this challenge requires an approach that considers the unique social, economic, and environmental realities of the Global South [66,67]. Proposed solutions should involve integrating grassroots movements into policy discussions, recognizing their contributions, and supporting them through robust legal and political frameworks that protect the environment while advancing health equity [68]. This includes promoting education, valuing local knowledge, and ensuring active community participation in sustainable development initiatives [69].

There is a pressing need to strengthen restorative justice projects, not only through reparation efforts but also by enabling meaningful dialogue among governments, local organizations, and international bodies. Such collaborations could facilitate the establishment of a shared understanding of long-term goals for environmental justice, addressing the health inequities that have emerged from decades of neglect [66,67].

In this context, one cannot overemphasize the importance of robust governance frameworks that combine effective legal protections with policies attuned to the lived experiences of vulnerable populations. This approach should not only deter environmental harm but also foster the development of sustainable industries capable of contributing to both local economies and environmental resilience [69]. By integrating these strategies and acknowledging the essential contributions of grassroots movements, the continent can take significant strides toward achieving a more equitable and sustainable future for all its communities.

## 4 Tying ethics to practical solutions

The lived realities of Africans underline that the vulnerability of impoverished populations is not merely an inconvenience—it is a matter of survival. The harsh reality of subsisting on less than $2.14 a day highlights the urgent need to address environmental justice and health equity, particularly in the Global South. These populations often endure the gravest consequences of the tragedy of the commons. How can justice and equity be attained?

John Rawls defines justice as fairness, emphasizing principles chosen under conditions of equality, such as equal basic liberties and the difference principle, which allows inequalities only if they benefit the least advantaged. His framework relies on the original position and the veil of ignorance to ensure impartiality, promoting fairness and equality in societal arrangements. Drawing from a Rawlsian perspective [70], the difference principle suggests that policies addressing climate change must prioritize benefiting those at the bottom of the economic ladder. Therefore, addressing the tragedy of the commons necessitates the collective management of shared resources to ensure that the most vulnerable populations are not disproportionately affected. At the same time, Rawlsian justice [70,71] advocates prioritizing the least advantaged—such as communities in oil-producing areas that suffer from environmental injustices, including oil spills and gas flaring. Mitigation strategies must prioritize the recovery and long-term well-being of these communities.

Millions of rural residents in Nigeria struggle with energy poverty, bearing the disproportionate burden of environmental degradation and pollution. For these communities, achieving both health equity and social justice requires access to sustainable energy and clean living environments. Utilitarian ethics supports immediate investments in renewable energy projects that address energy poverty while reducing the harmful impacts of fossil fuel reliance, thereby maximizing well-being for the majority [72].

The African philosophy of Ubuntu emphasizes the importance of community-driven decision-making processes [56], ensuring that all voices—particularly those most affected by environmental harms—are heard and considered in policy formation. The benefactors of the tragedy of the commons should not be the sole architects of policy. Voices from the Global South must be given meaningful participatory roles in the policymaking process. Hence this philosophy challenges the binary view of top-down versus bottom-up approaches by suggesting that ethically sound climate policy requires a fluid, inclusive model—one where international commitments are continuously integrated with grassroots priorities through sustained dialogue and shared accountability [[73], [74], [75]]. In countries such as Kenya and Malawi, community energy projects highlight the potential for decentralized energy governance, illustrating Ubuntu principles of communal benefit and solidarity. Kenya, Ethiopia, and Mozambique have provided off-grid solar energy solutions to rural communities [59]. However, ensuring these projects yield tangible social and environmental benefits remains essential [76,77]. Ubuntu helps navigate the ethical dilemma between immediate survival needs and long-term sustainability by advocating a human-centred approach. It prioritizes solutions that simultaneously improve immediate quality of life and ensure long-term ecological and social resilience [[78], [79], [80]].

Incorporating Ubuntu into international climate governance frameworks necessitates reimagining decision-making structures to prioritize authentic African participation, thus shifting from mere consultation to empowered negotiation [[73], [74], [75]]. From an Ubuntu perspective, existing international mechanisms—such as the Green Climate Fund—fall short by often imposing external solutions disconnected from local realities. Ubuntu argues for more participatory mechanisms that respect local knowledge, build trust, and sustain relationships over time [[78], [79], [80]].

Ubuntu, as complementary or superior to traditional Western ethical frameworks in addressing climate change and health inequities within the African context, emphasizes its relevance and cultural appropriateness [[78], [79], [80]]. While Rawls' difference principle emphasizes equity and prioritization of vulnerable populations, Ubuntu extends this further by grounding it in relational ethics—highlighting communal bonds, reciprocal responsibility, and collective resilience essential for long-term sustainability. Long term investments such as community empowerment and technology transfer are crucial for developing the skills and resources needed to thrive despite environmental degradation. By investing in local knowledge and capacity-building, affected populations are empowered to lead flourishing lives, addressing the root causes of health inequities. Incorporating these ethical frameworks into climate change and environmental justice efforts allows us to move beyond mere technical solutions, fostering approaches that are not only practical but also just, inclusive, and culturally relevant.

## 5 Recommendations

With the impending catastrophic impact of climate change on the vulnerable, there is a need to incorporate ethics into our recommendations and advocacy efforts related to climate change and health equity to ensure that policies and interventions prioritize the most vulnerable populations.

Fran Baum's *The New Public Health* explores the relationship between social determinants of health, such as education, and broader public health outcomes [81]. By addressing these determinants and advocating for justice, Baum’s recommendations demonstrate a commitment to ethical principles and practical solutions aimed at fostering a more equitable and sustainable future.

### 5.1 Education and advocacy along class lines

From an ethical perspective, it is crucial that education and advocacy efforts are not uniform but instead tailored to the specific realities faced by low-income groups, who are disproportionately affected by climate change. Education, particularly when analysed through the lens of socioeconomic class, plays a pivotal role in shaping health outcomes. Individuals from lower socioeconomic backgrounds frequently receive lower-quality education, which restricts their ability to comprehend and engage with public health information, including issues related to climate change. This disparity creates a gap in knowledge that exacerbates existing health inequities. Addressing these inequities necessitates confronting systemic barriers such as economic inequality and political disenfranchisement. Enhancing access to education—particularly in the domains of health and climate change—can strengthen advocacy efforts by empowering these populations to adopt health-promoting behaviours and participate in policy discourse. For such communication to be both effective and equitable, it must be attuned to socioeconomic divisions and the unique vulnerabilities experienced by diverse populations [82].

## 6 Integrate social determinants of health into climate and health policy

Policymakers must integrate social determinants of health into climate change and health policies. These policies should address how factors such as poverty, housing, and education contribute to adverse health outcomes, particularly in the context of climate change. From a Utilitarian perspective, this approach promotes the greatest good for the greatest number [83]. By addressing social determinants within climate and health policies, public well-being is maximized through the reduction of health inequities and increased resilience among disadvantaged groups. Such measures align with the ethical responsibility to enhance collective well-being.

## 7 Targeted advocacy and communication for vulnerable populations

Advocacy efforts should prioritize the design of strategic communication and educational programs tailored to the specific needs of vulnerable populations. These programs must include information about the health risks associated with climate change and practical steps to mitigate such risks. Ethical communication strategies must avoid further marginalizing or traumatizing vulnerable communities. From a Kantian perspective, advocacy should respect the inherent dignity of individuals by ensuring efforts are empowering rather than patronizing. This involves providing vulnerable populations with the tools and knowledge to safeguard their health without coercion [84,85].

## 8 Promote policies that address environmental injustices

Strengthening community-based education and advocacy initiatives aligns with the Ubuntu’s core philosophy of collective knowledge-sharing and mutual support by advancing localized ownership of climate-health initiatives rather than perpetuating dependency. Advocacy must support policies that address environmental injustices, particularly those disproportionately affecting low-income and rural communities, such as pollution and inadequate access to clean water and air. These efforts should also extend to supporting grassroots organizations working to advance environmental justice. Upholding the rights of all individuals to a healthy environment is a moral imperative [84,85]. Policies should embody this ethical obligation by ensuring marginalized communities are justly burdened by environmental harms and that their rights to clean air, water, and land are protected.

## 9 Build political will for action on health equity

Advocacy should aim to build political will to prioritize health equity in the context of climate change. This requires clearly articulating how health equity aligns with sustainable development goals and why addressing it is essential for the planet’s long-term health. Virtue ethics offers a compelling argument, as policymakers are encouraged to embody virtues such as justice, fairness, and responsibility [86,87]. By prioritizing health equity, they demonstrate moral integrity and a commitment to addressing the disproportionate burden of climate change on vulnerable populations.

### 9.1 Conclusion

The convergence of extreme weather, food insecurity, and fragile health systems demands ethical, inclusive, and context-sensitive responses. This paper has demonstrated how climate change deepens existing health inequities and highlighted the ethical imperatives—grounded in climate justice, distributive fairness, and the African philosophy of Ubuntu—for addressing these challenges.

Effective interventions must support grassroots leadership and be guided by both Rawlsian and Ubuntu ethics, promoting solidarity, equity, and shared responsibility. This calls for a shift in global climate governance from top-down approaches to frameworks rooted in mutual accountability and local empowerment.

Mitigating health impacts demands holistic frameworks integrating ethics, community engagement, education, and policy. Future research should move beyond descriptive accounts to evaluate community-led adaptation strategies, assess the long-term health effects of climate-induced displacement, and examine how ethical principles are integrated into national climate policies. Empirical studies are also needed to inform the ethical redesign of international financing mechanisms, ensuring they build resilience and restore agency in the most affected regions. Bridging ethical theory with practical action is essential to achieving a climate-resilient and health-equitable future for Africa.

## CRediT authorship contribution statement

**Adetayo Emmanuel Obasa:** Writing – review & editing, Writing – original draft, Validation, Investigation, Conceptualization.

## Declaration of competing interest

I do not have a COI nor DoCI to declare.
